# Landauer Bound in the Context of Minimal Physical Principles: Meaning, Experimental Verification, Controversies and Perspectives

**DOI:** 10.3390/e26050423

**Published:** 2024-05-15

**Authors:** Edward Bormashenko

**Affiliations:** Department of Chemical Engineering, Biotechnology and Materials, Engineering Sciences Faculty, Ariel University, Ariel 407000, Israel; edward@ariel.ac.il; Tel.: +972-074-729-68-63

**Keywords:** Landauer principle, entropy, Abbe limit, Margolus–Levitin limit, Bekenstein limit, Planck–Boltzmann time, Szilárd engine

## Abstract

The physical roots, interpretation, controversies, and precise meaning of the Landauer principle are surveyed. The Landauer principle is a physical principle defining the lower theoretical limit of energy consumption necessary for computation. It states that an irreversible change in information stored in a computer, such as merging two computational paths, dissipates a minimum amount of heat $k_{B}Tln2$ per a bit of information to its surroundings. The Landauer principle is discussed in the context of fundamental physical limiting principles, such as the Abbe diffraction limit, the Margolus–Levitin limit, and the Bekenstein limit. Synthesis of the Landauer bound with the Abbe, Margolus–Levitin, and Bekenstein limits yields the minimal time of computation, which scales as $\tau_{min}\sim \frac{h}{k_{B}T}$. Decreasing the temperature of a thermal bath will decrease the energy consumption of a single computation, but in parallel, it will slow the computation. The Landauer principle bridges John Archibald Wheeler’s “it from bit” paradigm and thermodynamics. Experimental verifications of the Landauer principle are surveyed. The interrelation between thermodynamic and logical irreversibility is addressed. Generalization of the Landauer principle to quantum and non-equilibrium systems is addressed. The Landauer principle represents the powerful heuristic principle bridging physics, information theory, and computer engineering.

## 1. Introduction

The Landauer principle is one of the limiting physical principles that constrains the behavior of physical systems. There exist fundamental laws and principles setting the limits of physical systems. These laws do not predict or describe the behavior of physical/engineering systems but limit or restrict their functioning. A realistic natural/engineering system can only provide limited functionalities because its performance is physically constrained by some basic principles [1]. Some of these limits are engineering ones. For example, a key engineering bottleneck for the development of new generations of computers today is integrated circuit manufacturing, which confines billions of semi-conducting units in several ${\mathrm{cm}}^{2}$ of silicon with extremely low defect rates [2]. Another engineering constraint is imposed by limits on individual interconnects [2]. Despite the doubling of the transistor density according to the Moore law, semiconductor integrated circuits would not operate without fast/dense interconnects. Metallic wires can be either fast or dense but not both at the same time—a smaller cross-section increases electrical resistance, while a greater height or width increases parasitic capacitance with neighboring wires (wire delay grows with RC) [2]. Other constraints limiting the operation of physical (natural or engineering) systems are fundamental ones, and they emerge from the deepest foundations of physics. Limiting physical principles appeared in physics relatively late. It seems that the first limiting principle historically was the Abbe diffraction limit, discovered in 1873, which states that in light with wavelength λ λ, traveling in a medium with refractive index *n* and converging to a spot with half-angle θ θ, θ will have a minimum resolvable distance of *d*, as supplied by Equation (1):

(1) $$d=\frac{\lambda }{2nsin\theta },$$

where the minimum resolvable distance *d* is defined as the minimum separation between two objects that results in a certain level of contrast between them [3,4]. The Abbe diffraction limit is the maximum resolution possible for a theoretically perfect, or ideal, optical system [3,4]. Thus, it is not the engineering but the fundamental physical principle. The Abbe diffraction limit arises from the idea that the image arises from a double diffraction process [3,4]. Other diffraction limit formulae, known as the Rayleigh and Sparrow limits, were suggested [3,4]. These formulae coincide with the Abbe limit within a numerical coefficient; thus, the value of the numerical multiplier appearing in Equation (14) is not exact [3,4,5].

In spite of the fact that the Abbe diffraction limit is rooted in classical physics, the role of the limiting principles in the realm of classical physics is more than modest. The situation has changed dramatically within modern physics. In relativity, the speed of light in a vacuum, labeled *c*, is a universal physical constant of ca. 300,000 km per second, and according to the special theory of relativity, *c* is the upper limit for the speed at which conventional matter or energy (and, consequently, any signal carrying information) can travel through space [6,7]. It is impossible for signals or energy to travel faster than *c*. The speed at which light waves propagate in a vacuum is independent of both the motion of the wave source and the inertial frame of reference of the observer, thus enabling the Einstein synchronization procedure for clocks [6,7]. The limiting status of the speed of light in a vacuum was intensively disputed in the last few decades, and theories assuming a varying speed of light have been proposed as an alternative way of solving several standard cosmological problems [8,9]. Recent observational hints that the fine structure constant may have varied over cosmological scales have given impetus to these theories [8,9]. Theories in which the speed of light traveling in a vacuum appeared as an emerging physical value were suggested [9]. We adopt unequivocally the limiting status of the speed of light in a vacuum *c* and demonstrate that this status generates other limiting physical principles, and just this status gives rise to consequences emerging from the Landauer principle.

The main limiting principle of quantum mechanics is the Heisenberg uncertainty principle. It states that there is a limit to the precision with which certain pairs of physical properties, such as position *x* and momentum *p* (or time *t* and energy *E*), can be simultaneously measured. In other words, and more accurately speaking, when one property is measured, the less accurately the other property can be established (see Equations (2) and (3)):

(2) $$\sigma_{x}\sigma_{p}\geq \frac{\hbar }{2},$$

(3) $$\sigma_{t}\sigma_{E}\geq \frac{\hbar }{2},$$

where $\sigma_{x},\sigma_{p},\sigma_{t}$, and $\sigma_{E}$ are standard deviations of the position, momentum, time, and energy, respectively, and $\hbar =\frac{h}{2\pi }$ is the reduced Planck constant [10,11]. The time–energy uncertainty principle, supplied by Equation (3), needs more detailed discussion to be supplied in the context of the Mandelstam–Tamm and Margolus–Levitin bounding principles.

The limiting value of the light propagating in a vacuum *c* combined with the Heisenberg uncertainty principle together yield the Bremermann limit, which supplies a limit on the maximum rate of computation that can be achieved in a self-contained system [12]. The Bremermann limit is derived from Einstein’s mass–energy equivalency and the Heisenberg uncertainty principle, and is $\frac{c^{2}}{h}≅1.35\times 10^{50}$ bits per second per kilogram of the computational system [12]. Consider that the Bremermann limit is built of the fundamental physical constants only.

Quantum mechanics also gives rise to the Mandelstam–Tamm and Margolus–Levitin limiting principles [13,14]. The Mandelstam–Tamm quantum speed limit states the time it takes for an isolated quantum system to evolve between two fully distinguishable states, as given by Equation (4):

(4) $$\tau >\tau_{MT}=\frac{h}{4\Delta E},$$

where ΔE is the energy uncertainty. The Margolus–Levitin limiting principle supplies a surprising result, predicting the maximum speed of dynamic evolution of the system [15]. The Margolus–Levitin limiting principle supplies the minimum time it takes for the physical system to evolve into an orthogonal state (labeled $\tau_{⊥})$. It should be emphasized that this minimum time $\tau_{⊥}$ depends only on the system average energy minus its ground state (denoted $E-E_{0}$, and not on the energy uncertainty ΔE, as follows from Equation (4) [15]. To simplify the formulae, we chose the zero-level energy in such a way that $E_{0}=0$ so that the Margolus–Levitin limiting principle yields for the minimal time bound, denoted as $\tau_{ML}$ in Equation (5):

(5) $$\tau_{⊥}>\tau_{ML}=\frac{h}{4E}$$

Another important fundamental limiting principle is supplied by the Bekenstein bound [16]. Bekenstein demonstrated that there exists a universal upper bound of the entropy-to-energy ratio $\frac{S}{E}$ for an arbitrary system confined by radius *R*, and this limit is expressed by $\frac{S}{E}=\frac{2\pi R}{\hbar c}$ [16]. In other words, the Bekenstein bound defines an upper limit on the entropy *S*, which can be confined within a given finite region of space that has a finite amount of energy *E*, or conversely, the maximum amount of information required to perfectly describe a given physical system with a given, fixed energy *E* down to the quantum level [16]. The bound value of entropy *S* is given by Equation (6):

(6) $$S\leq \frac{2\pi k_{B}RE}{\hbar c},$$

where *R* is the radius of a sphere that can enclose the given system, and *E* is the total mass–energy including any rest masses [16]. We will discuss below the Margolus–Levitin and the Bekenstein bounds in their relation to the Landauer principle.

## 2. Results

### 2.1. What Is Information? The Meaning of the Landauer Principle

What is information? The ambiguity of the notion of information hinders the physical interpretation of this notion. Numerous definitions of information were suggested [17,18]. I am quoting from Ref. [17]: “Information can be data, in the sense of a bank statement, a computer file, or a telephone number. Data in the narrowest sense can be just a string of binary symbols. Information can also be meaning” [17]. Informational theory is usually supplied in a pure abstract form that is independent of any physical embodiment. Intellectual breakthrough in the mathematization of information is related to the pioneering works by Claude Shannon, who introduced the information entropy of a random variable understood as the average level of “information” or “uncertainty” inherent to the variable’s possible outcomes [19,20]. Given a discrete random variable X Ψ, which takes values in the alphabet Ψ, X and is distributed according to p:Ψ→[0,1] *p*: X → [0, 1], the Shannon measure of information/Shannon entropy, denoted as H(Ψ), is given by Equation (7):

(7) $$H\left(\Psi \right)=-\sum_{x\in \Psi }p\left(x\right)logp\left(x\right)$$

The Shannon measure of information is a very general mathematical concept, and regrettably, it is often mixed in the literature with thermodynamic entropy [21,22,23,24,25]. A distinction for the Shannon measure of information is made in Refs. [21,22,23,24,25]. Again, the Shannon measure of information is a very useful mathematical concept completely disconnected from the process of recording information, information carrier material, reading, and erasing information.

**Figure 1:**
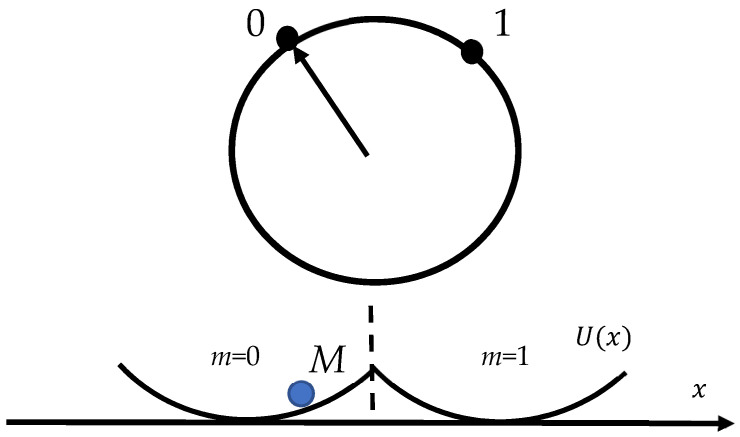
Particle *M* placed in the twin-well potential is depicted. The position of the particle in the double-well potential will determine the state of the single bit. If the particle is found on the left-hand side of the potential, then we say that the bit is in the “zero” state. If it is found on the right-hand side of the well, then we define that the bit is in the “one” state. The picture is taken from the Bormashenko Ed. “Generalization of the Landauer Principle for Computing Devices Based on Many-Valued Logic” [35].

In contrast, Rolf Landauer, in his pioneering and fundamental papers published in 1961–1996, argued that information is physical and has an energy equivalent [25,26,27,28,29]. It may be stored in physical systems such as books and memory chips and is transmitted by physical devices exploiting electrical or optical signals [26,27,28,29]. Indeed (I am quoting from Ref. [29]), “computation, whether it is performed by electronic machinery, on an abacus or in a biological system such as the brain, is a physical process. It is subject to the same questions that apply to other physical processes: How much energy must be expended to perform a particular computation? How long must it take? How large must the computing device be? In other words, what are the physical limits of the process of computation?” If we adopt the idea that computation is a physical process, it must obey the laws of physics, and first and foremost the laws of thermodynamics [26,27,28,29]. This thinking leads to the new limiting physical principle, which establishes the minimum energy cost for the erasure of a single memory bit for the system operating at the equilibrium temperature *T*. This is exactly the Landauer principle. The Landauer principle may be derived in different ways; we start from the one-bit system depicted schematically in Figure 1. The picture depicts the Brownian particle *M* confined within a double-well potential, shown in Figure 1 and addressed in detail in Refs. [27,28,29,30]. When the barrier is much higher than the thermal energy, the Brownian particle will remain in either well (left or right) for a long time. Thus, the particle located in the left or right well can serve as the stable informational states “0” and “1” of a single information bit (the informational states are denoted *m* = 0 and *m* = 1 in Figure 1, where *m* is the parameter, characterizing the statistical state of the double-well system). The average work *W* necessary to switch the statistical state of a memory under the isothermal process from the state *Ψ* with distribution $p_{m}$ to $\Psi^{\prime }$ with distribution $p_{m}^{\prime }$ is given by Equation (8):

(8) $$W\geq F\left(\Psi^{\prime }\right)-F\left(\Psi \right),$$

where F(Ψ) is the Helmholtz free energy of the system supplied by Equation (9):

(9) $$F\left(\Psi \right)=\sum_{m}p_{m}F_{m}-k_{B}TH\left(\Psi \right)=\sum_{m}p_{m}F_{m}+k_{B}T\sum_{m}p_{m}lnP_{m},$$

where $F_{m}=E_{m}-TS_{m}$ is the Helmholtz free energy of the conditional states, and $H\left(\Psi \right)=-\underset{m}{\sum }p_{m}lnp_{m}$ is the Shannon entropy of the informational states, in the Shannon entropy of the informational states, which equals to their entropies $S_{m}$ [21,22,23,24,25,30]. For a symmetrical well and a random bit $p_{0}=p_{1}=\frac{1}{2}$, we immediately obtain the Landauer bound, supplied by Equation (10):

(10) $$W=k_{B}Tln2$$

The exact meaning of Equation (10) supplies the energy necessary for resetting/erasing one random bit stored in a symmetric memory unit [30]. For asymmetric memory units, $\Delta F_{reset}$ is not necessarily equal to $-k_{B}TH\left(\Psi \right)$ and the limiting Landauer principle is given by the following inequality:

(11) $$W_{reset}\geq \Delta F_{reset}$$

The exact equality is attained if the reset is thermodynamically reversible [30]. This does not contradict the logical irreversibility of the reset, which implies that the entropy H(Ψ) of the informational states decreases [30,31]. It is noteworthy, that the Landauer bound, given by Equation (10), is related only to a single information-bearing degree of freedom of the entire computing system.

The relation between logic and thermodynamic reversibility will be discussed below. Again, the energetic cost on one random bit is supplied by the limiting physical principle, expressed by Equation (11). A detailed discussion of Equations (10) and (11) is supplied in Ref. [30]. An accurate and rigorous derivation of Equations (10) and (11) emerging from microscopic reasoning is supplied in Ref. [32]. We again consider the particle in the twin-well potential *U*(*x*), shown in Figure 1. We assume that before the erasure we want half of the bits to be in the ‘‘one’’ state and the other half to be in the ‘‘zero’’ state. We also adopt the idea that the ensemble of bits is in contact with a thermal reservoir where the temperature of the reservoir *T* is low enough not to change the state of the bits; in other words, $k_{B}T<\Delta U$ takes place [32]. The system will instead reach a ‘‘local’’ thermal equilibrium in one of the half-wells. We therefore assume that the initial statistical state is described by the following for the bits before erasure (see Figure 1):

(12) $$\rho_{in}\left(x,p\right)=\frac{1}{Z}exp\left\{-\beta \left[U\left(x\right)+\frac{p^{2}}{2M}\right]\right\}$$

whereas after the erasure, the distribution function is given by Equation (13):

(13) $$\rho_{fin}\left(x,p\right)=\left\{\begin{array}{c} \frac{2}{Z}exp\left\{-\beta \left[U\left(x\right)+\frac{p^{2}}{2M}\right]\right\},\;for\;x>0\\ 0,\;\mathrm{for}\;x<0 \end{array}\right\},$$

where *x* is the position, *p* is the momentum of the particle *M*, $\beta =\frac{1}{k_{B}T}$, and $Z=\int exp-\left\{\left[U\left(x\right)+\frac{p^{2}}{2m}\right]\beta \right\}dpdx$ is the partition function [33,34]. After the routine transformations, it is demonstrated that to erase one bit of information, on average, the work performed on the system has to be equal to or greater than $ln2k_{B}T$, or, equivalently, that the heat dissipation by the system into the heat reservoir has to be greater than or equal to the Landauer bound $ln2k_{B}T$ [32]. Generalization of the Landauer principle for computing devices based on many-valued logic (*N*-based logic), exploiting *N* identical potential wells, was reported [30,35]. The energy necessary for the erasure of one bit of information (the Landauer limiting bound) $W=k_{B}Tln2$ remains untouched for computing devices exploiting many-valued logic [30,35].

### 2.2. The Landauer Limit and the Margolus–Levitin Limiting Principle

Now we are ready to combine the Landauer bound with the Margolus–Levitin limiting principle, given by Equation (5). Consider the computing unit, based on the physical device for which the Landauer limiting principle is true (the device exploiting identical potential wells confining the particle may be taken as an example) [30,32,35]. This device operates in a thermal equilibrium with its surroundings (thermal bath), which is kept at a constant temperature *T*. Let us pose the following question: What is the minimal time it will take for this device to make a single computing operation? Assuming in Equation (5) that $E≅k_{B}T\ln2$, we obtain the following very rough estimation for the minimum “Margolus–Levitin–Landauer” time necessary for a single computation, denoted as $\tau_{MLL}$:

(14) $$\tau_{MLL}\geq \frac{h}{4\ln2k_{B}T}=\frac{\tau_{PB}}{4ln2}$$

where $\tau_{PB}=\frac{h}{k_{B}T}$ is the Planck–Boltzmann thermalization time, which is conjectured to be the fastest relaxation timescale for thermalization of the given system [36]. We assume in Equation (14) that that the energy cost of a single computation equals the energy necessary for the transfer of the system into the orthogonal quantum state. Again, we choose the zero-level energy in such a way that $E_{0}=0$ [15]. The numerical multiplier appearing in Equation (14) should not be taken too seriously. The values of these multipliers are not exact when the limiting physical principles are considered, as already mentioned when the Abbe diffraction limit (see Equation (1)) was discussed. It is noteworthy that the Margolus–Levitin–Landauer time given by Equation (14) is independent of the geometrical dimensions of the computing unit. Formula (14) may be called the Margolus–Levitin–Landauer bound. The Planck–Boltzmann thermalization time should not be mixed up with the Planck time, which is the time span at which no smaller meaningful length can be validly measured due to the indeterminacy expressed in Werner Heisenberg’s uncertainty principle. 

Let us estimate now the Landauer time for the ambient conditions. Assuming $h≅6.626\times 10^{-34\;}\mathrm{Js},\;k_{B}≅1.38\times 10^{-23}\frac{J}{K},\;T≅300\;K$, we calculate $\tau_{MLL}≅0.9\times 10^{-11}\sim 10^{-11}\;s.$ Thus, a single computing unit may perform not more than $10^{11}$ erasures per second in ambient conditions. 

Other approaches for the bounds of the finite time computation were suggested [37,38,39,40,41]. For a slowly driven (quantum) two-level system weakly coupled to a thermal bath, the finite-time Landauer bound takes the simple form supplied by Equation (15):

(15) $$W\geq k_{B}T\left(ln2+\frac{\pi^{2}}{4\Gamma \tau }\right)+O\left(\frac{1}{\Gamma^{2}\tau }\right),$$

where *τ* is the total time of the computation process and *Γ* is the thermalization rate. It should be emphasized that all of the approaches suggest the emergence of the Planckian thermalization time scale $\tau_{PB}=\frac{h}{k_{B}T}$ (we denote it as the Planck–Boltzmann time) as the shortest timescale for information erasure, as also immediately follows from the Margolus–Levitin–Landauer bound supplied by Equation (14) (see Ref. [41]). Finite-size corrections to the Landauer bound are reported in Ref. [42]. Equations (14) and (15) supply the trade-off important for development of the computing devices. Engineers want computing devices to be as energy efficient as possible; thus, they try to diminish the energy necessary for a single computation [43]. It should be emphasized that the Landauer limit establishing the minimal energy cost $W=k_{B}Tln2$ for a single erasure operation emerges from the equilibrium thermodynamic considerations, and it is independent of the engineering realization of the computing unit [43]. However, this decrease in the energy cost of computation due to a decrease in the temperature *T* inevitably results in an increase in a single computation time, as follows from the Margolus–Levitin–Landauer bound supplied by Equation (14).

### 2.3. The Landauer Limit and the Bekenstein Bound

Now we find ourselves in the realm of relativity. We will demonstrate that the Bekenstein bound [16] also restricts the computation time. Consider a computational unit with a characteristic dimension of *R*. Cum grano salis we assume that the minimal time of the single computation (we call it the Bekenstein time and denote it as $\tau_{B})$ is given by $\tau_{B}≅\frac{R}{c}$, which is the minimal time possible for the transfer of the particle from one half of the double-well potential to another one. Now we address Equation (6). The entropy change necessary for erasing 1 bit of information is estimated as $S=k_{B}ln2$. According to the Landauer principle $E≅k_{B}Tln2$, substituting $\tau_{B}≅\frac{R}{c}$, $S=k_{B}ln2$, and $E≅k_{B}Tln2$ yields Equation (16):

(16) $$\tau_{B}\geq \frac{h}{{\left(2\pi \right)}^{2}k_{B}T}=\frac{\tau_{PB}}{{\left(2\pi \right)}^{2}},$$

It is immediately recognized that the Planck–Boltzmann thermalization time appears as a single time scale in the eventual bound, supplied by Equation (16). This time scale is independent of the dimensions of the computing unit. Comparing Equation (16) to Equation (14) yields $\tau_{B}<\tau_{MLL};$ however, the values of these time scales are close one to another. It is seen that the Landauer limiting principle allows for fundamental ideas emerging from relativity and quantum mechanics to be unified. The minimal times of computation arising from the Margolus–Levitin and Bekenstein bounds are close to one to another. Thus, the Landauer principle in a certain sense bridges relativity and quantum mechanics. This idea will be discussed below. It should be emphasized the Landauer principle holds for a variety of quantum systems [39,44,45,46,47,48,49].

### 2.4. The Abbe Diffraction Limit and the Landauer Principle

**Figure 2:**
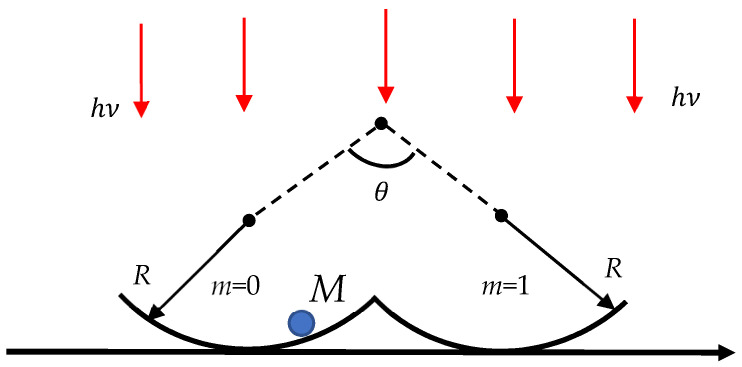
A twin-well system containing particle *M* illuminated with monochromatic light ν is depicted. The system is in thermal equilibrium with the surrounding *T*.

Now we address the Abbe diffraction limit (see Equation (1)) discussed in Section 1 and addressed in detail in the classic textbooks devoted to optics [3,4]. Consider the twin-well computational system depicted in Figure 2 and representing particle *M* confined within the twin-well potential. We use the monochromatic light ν, λ (ν is a frequency, λ is a wavelength) for identification of the particle location. According to Equation (1), the identification of the particle location is still possible when λ≅2dnsinθ≅4Rnsinθ takes place, where *n* is the refractive index and angle θ is shown in Figure 2. If the same monochromatic light beam ν, λ is used for the erasure of information, i.e., for the transfer of the particle from one half-well to another, and the Landauer principle is adopted, we estimate $h\upsilon =h\frac{c}{\lambda }≅k_{B}Tln2$, where *c* is the light speed. Thus, we obtain $\lambda ≅\frac{hc}{ln2k_{B}T}$. The minimum time necessary for a single computation is roughly estimated as $\tau_{min}≅\frac{2R}{c}$. Combining these estimations yields the minimum time of a computation:

(17) $$\tau_{min}≅\frac{1}{2nsin\theta ln2}\frac{h}{k_{B}T}$$

The minimum computation time corresponding to n≅1, $\theta =\frac{\pi }{2}$ is estimated as follows:

(18) $$\tau_{min}≅\frac{1}{2ln2}\frac{h}{k_{B}T}≅\tau_{PB}$$

Again, the minimum time span of computation scales as the Planck–Boltzmann thermalization time, independent of the geometrical parameters of the system, given by $\tau_{PB}=\frac{h}{k_{B}T}$.

### 2.5. Breaking the Landauer Limit

It should be emphasized that derivation of the Landauer bound emerging from the analysis of the behavior of the particle placed in the twin-well potential, shown in Figure 1 and Figure 2, implies the symmetrical configuration of the potential [30,32]. In the asymmetrical twin-well potential the Landauer bound may be broken [31,50,51]. The Landauer principle for information erasure is valid for a symmetric double-well potential but not for an asymmetric one. Physically, the reduced work arises when the starting state is not in equilibrium, and other degrees of freedom do work that compensates for the work required to erase. More simply, erasing from a small well to a large well transfers a particle from a small box to a larger one but never the reverse [51].

### 2.6. The Landauer Principle and Thermodynamics of Small Systems

**Figure 3:**
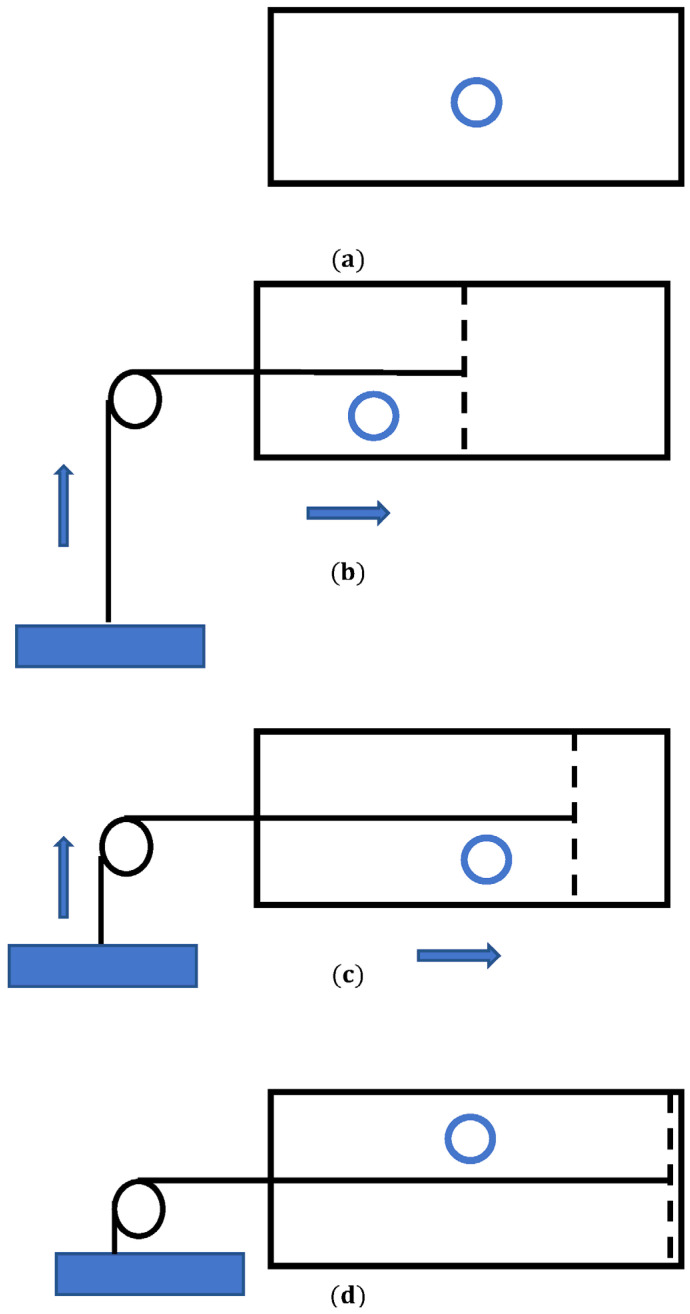
A scheme of the Leo Szilárd minimal engine is depicted. (**a**) A particle in a box is shown; (**b**) the partition defines the location of the particle; (**c**) the particle pushes the piston and the engine performs work; (**d**) one bit of information is erased.

The Landauer principle may be understood in the context of the minimal thermal engine suggested by Leo Szilárd in 1929 [52]. Leo Szilárd is famous for his letter with Albert Einstein’s signature that resulted in the Manhattan Project. In Leo Szilárd’s original formulation, the engine exploits single-molecule gas confined in a box of volume $V_{1}$ contacting a thermal bath at temperature *T*, as depicted in Figure 3. As in any other thermal engine, the molecule/particle pushes the piston and the engine performs work (say, lifting a load, as shown in Figure 3b,c). Thus, the Szilárd energy transforms heat collected from the bath in the task, being the minimum thermal engine [52]. We are interested in the informational interpretation of the Szilárd engine, which is closely related to the Landauer principle.

Consider the location of the particle within a box. Divide the box into two equal parts. Actually, the information concerning which side the molecule is in after dividing the box can be utilized to extract work, e.g., via an isothermal expansion, under T=const. Let us explain this idea: Isothermal expansion of the single-molecule gas from volume $V_{1}$ to volume $V_{2}$ followed by the motion of the piston yields the work, given by $=k_{B}Tln\frac{V_{2}}{V_{1}}$. In this particular case, the box is divided into two equal halves: $V_{2}=2V_{1}$ and $W=k_{B}Tln2$. However, this result may be interpreted in terms of the information theory. Indeed, after the expansion we lost the information about the precise location of the particle. Thus, we performed erasure of one bit of information. In other words, we converted one bit of information into the work $W=k_{B}Tln2$. This is exactly the Landauer bound [26,27,28,29]. Instead of displacement of the piston, we may imagine the Maxwell demon, which introduces or pulls out the impermeable partition that fixes/erases the location of the particle. Thus, it turns out that the Landauer principle is closely related to the famous Maxwell demon paradox [53].

It seems that the action of the Szilárd engine contradicts the second law of thermodynamics. Indeed, let us make the Szilárd engine cyclic. To return the initial state, the partition/piston can be removed without any work consumption, and the whole process can be repeated in a cyclic manner. All thermodynamic processes are defined as isothermal and reversible [53]. This engine apparently violates the Kelvin–Planck statement of the second law (and it is well known that it is actually equivalent to the Clausius and Carnot formulations) by converting heat directly into an equivalent amount of work through a cyclic process [53]. Now it is generally accepted that the measurement process including erasure or reset of the Maxwell demon memory requires a minimum energy cost of at least $W=k_{B}Tln2$, associated with the entropy decrease of the engine, and that it saves the second law. A quantum Szilárd engine was addressed [53,54]. A demonless quantum Szilárd engine was studied [53]. It was demonstrated that the localization holds the key along with the Landauer principle to save the second law and presents a complementary resolution of the quantum version of Szilárd’s paradox [53]. Quantum mechanics-rooted arguments are necessary for the justification of the third law of thermodynamics. Quantum mechanics also saves the second law, suggesting that quantum mechanics has strong ties in the foundations of thermodynamics and information theory [53].

Numerous questions related to the information interpretation of the Szilárd engine remain open. However, it is clear that the Szilárd engine links the Landauer principle to the thermodynamics of small systems, which was rapidly developed in the past decade [54,55,56,57]. For example, it will be instructive to address the minimal (single-particle) Carnot engine, exploited for the erasure of information in heat baths [58]. It is noteworthy that the efficiency of the minimum Carnot is given by the traditional Carnot expression when the motion of gas particles is temporally averaged (instead of the usual spatial averaging) [58]. Only a few experimental realizations of the Szilárd engine have been reported [59,60,61,62]. A single-electron box operated as a Szilárd engine enabled the extraction of $k_{B}Tln2$ heat from the reservoir at temperature *T* per one bit of created information [59]. The information was encoded in the position of an extra electron in the box [59]. 

### 2.7. The Landauer Principle and the “It from Bit” Archibald Wheeler Paradigm 

In 1989, John Archibald Wheeler suggested the global concept aphoristically called “it from bit.” “It from bit” symbolizes the idea that every item in the physical world has at the bottom—at a very deep bottom, in most instances—an immaterial source and explanation, that what we call reality arises in the last analysis from the posing of yes–no questions and the registering of equipment-evoked responses—in short, that all things physical are information-theoretic in origin and that this is a participatory universe. Three examples may illustrate the theme of “it from bit.” First is the photon. With a polarizer over the distant source and an analyzer of polarization over the photodetector under watch, we ask the yes or no question, “Did the counter register a click during the specified second?” If yes, we often say, “A photon did it.” We know perfectly well that the photon existed neither before the emission nor after the detection. However, we also have to recognize that any talk of the photon “existing” during the intermediate period is only a blown-up version of the raw fact, a count. The yes or no that is recorded constitutes an unsplittable bit of information. A photon, Wootters and Zurek demonstrate, cannot be cloned [63]. Actually, the Landauer principle fills the “it from bit” idea with physical content when supplying the link between “information” and physically measurable properties of real systems. This bridge was built in a series of recent papers [64,65,66,67,68,69,70,71,72,73,74,75,76,77]. The principle of mass–energy–information equivalence, which proposes that a bit of information is not just physical, as already demonstrated, but also has a finite and quantifiable mass while it stores information, was suggested [64,65,66,67,68,69,70,71,77]. According to Herrera, a change to one bit of information (provided the temperature is fixed) leads to a decrease in the mass of the system by an amount whose minimal value is [64]:

(19) $$\Delta M=\frac{k_{B}T}{c^{2}}ln2$$

It is noteworthy that the Landauer principle in Ref. [64] is called “Brillouin’s principle”. Indeed, the idea that the dissipation of energy associated with a change to one bit of information is a fundamental process independent of the technicalities associated with information processing (regarded today as the Landauer principle) first appears in the work by Leon Brillouin [65].

The idea that mass may be ascribed to information was developed in Refs. [66,67,68,69,70,71,72,73,74,75]. According to Vopson, an equivalent mass of excess energy is created in the process of lowering the information entropy when a bit of information is erased, and vice versa. Once a bit of information is created, it acquires a finite mass, denoted as $m_{bit}$ [66]. Using the mass–energy equivalence principle, the mass of a bit of information is given by Equation (20) (compare it to Equation (19)) [66]:

(20) $$m_{bit}=\frac{ln2k_{B}T}{c^{2}}$$

The idea that a mass may be ascribed to a bit of information was criticized recently, as will be mentioned below. The mass of a bit of information at room temperature calculated with Equation (20) (*T* = 300 K) is $3.19\times 10^{-38\;}\mathrm{kg}$, as estimated in Ref. [66]. Now consider the particle with energy *E* in contact (not necessarily in thermal equilibrium) with a thermal bath *T*. The energy of the particle may be used for erasing information within the thermal bath. The maximum information (as measured in bits), denoted as $I_{max}$, which may be erased by the particle in contact with the bath, according to the Landauer principle, equals: [75]:

(21) $$I_{max}=\frac{E}{k_{B}Tln2}=\frac{mc^{2}}{k_{B}Tln2},$$

where *m* is the relativistic mass of the particle. The value $I_{max}$ may be seen within the Landauer context as the maximum informational content of a relativistic particle. If the potential energy of the particle is negligible and $\frac{v}{c}\ll 1$ is adopted (*v* is the velocity of the particle), Equation (21) is re-written as follows [75]:

(22) $$I_{max}=\frac{m_{0}c^{2}}{k_{B}Tln2}$$

The value $I_{max}$ supplied by Equation (22) may be understood as the maximum informational content of a particle *at rest* [75]. The particle may exchange information with the medium, if *at least one bit* of information is erased in medium by the particle; thus, inequality $I_{max}\geq 1$ should hold. This inequality yields:

(23) $$m_{0}\geq \frac{k_{B}Tln2}{c^{2}}$$

A particle with a rest mass smaller than ${\tilde{m}}_{0}=\frac{k_{B}Tln2}{c^{2}}$ will not erase information in the medium at the temperature *T*. Assuming T=2.73 K (which is the temperature of the cosmic microwave background [78]), we obtain the estimation ${\tilde{m}}_{0}≅\frac{1.6\;\times \;10^{-4\;}\mathrm{eV}}{c^{2}}≅2.0\times 10^{-40}\;\mathrm{kg}$. It should be emphasized that all of the elementary particles known today (including neutrino $m_{neutrino}<0.120\frac{eV}{c^{2}}$) are heavier than ${\tilde{m}}_{0}=2.0\times 10^{-40}\mathrm{kg}$. Particles lighter than ${\tilde{m}}_{0}=2.0\times 10^{-40}\mathrm{kg}$ will not transform the information to the universe and are expected to be undetectable.

The Landauer principle enables the estimation of the computational capacity of the entire universe, which is large but finite [66,76,79,80]. We denote the total informational capacity of the universe as $I_{tot}$, which may be estimated as follows:

(24) $$I_{tot}=\frac{m_{tot}c^{2}}{k_{B}T},$$

where $m_{tot}=1.5\times 10^{53}\;\mathrm{kg}$ is the mass of the observable universe [81]. Substituting and T=2.73 K , we obtain $I_{tot}≅3.0\times 10^{92}\;\mathrm{bits}$, which is in a satisfactory vicinity to the estimation reported in Ref. [80], which was based on quite different considerations.

The Landauer minimum principle enables a fresh glance at the famous “dark matter” problem [82,83,84]. Dark matter is the mysterious substance that dominates the mass budget of the universe from sub-galactic to cosmological scales, which is arguably one of the greatest challenges of modern physics and cosmology [82,83,84]. We still do not know how to explain how stars orbit in galaxies or how galaxies orbit in clusters. A wide array of candidates for particle dark matter was suggested, including thermal relics (WIMPs), neutralinos, and sterile neutrinos [83,84,85,86]. However, numerous experiments have failed to find evidence for the suggested dark matter particles, and it was hypothesized that gravity theory should be modified [87]. Equation (23), emerging from the Landauer minimum principle, enables revisiting the “dark matter” problem [66,75]. Indeed, if dark matter is built from the particles for which $m<{\tilde{m}}_{0}≅\frac{k_{B}Tln2}{c^{2}}≅2.0\times 10^{-40}\;\mathrm{kg}$ takes place, they could not be registered due the fact that they do not transform information to the surrounding media and experimental devices [66,75]. 

Let us continue thinking within the Wheeler “it from bit” paradigm. The Landauer minimum principle supplies a new glance at the problem of the great unification of physics. Equation (21) may be easily extended to fields. Consider a field (for example, an electromagnetic field) in a thermal contact (not necessarily in thermal equilibrium, as it takes place in a black body radiation problem) with a surrounding/thermal bath *T*. The energy of the field may be used for isothermal erasing of information in the surroundings. The maximum information to be erased by the field (seen as the informational content of the field) according to the Landauer principle is given by:

(25) $$I_{max}=\frac{E_{f}}{k_{B}Tln2}$$

where *E_f_* is the energy of the field. It is noteworthy that the physical nature of the field does not matter. If the information and the temperature are taken as basic physical quantities, Equation (25) will be universal for all kinds of physical fields. The field is capable of isothermally erasing the information if the bounding inequality $E_{f}>k_{B}Tln2$ is true. The Landauer principle changes the status of the temperature, usually seen as the derivative of basic physical quantities such as energy and entropy [33,34]. Contrastingly, the Landauer principle tells us that it is just the temperature that determines the possibility of erasing/recording the information, seen as a basic physical value [88]. 

### 2.8. Experimental Verification of the Landauer Principle

Landauer bound was tested in a series of experimental investigations [46,59,87,88,89]. Koski et al. tested the Landauer principle with the minimum Szilárd engine (see Section 2.6 and Figure 3) [52,59]. The main element the Szilárd engine was the single-electron box (abbreviated SEB) [59], which consisted of two small metallic islands connected by a tunnel junction [59]. The SEB was maintained at a dilution-refrigerator temperature in the 0.1 K range. The authors provided an experimental demonstration of extracting nearly $k_{B}Tln2$ of work for one bit of information, in accordance with the Landauer principle [59]. Use of the trapped ultra-cold ion enabled the demonstration of a quantum version of the Landauer principle in the experimental study by Yan et al. [46]. Ref. [89] reported experimental testing of the Landauer bound at low values of $k_{B}T$. The authors demonstrated that for the logically reversible operations, energy dissipations much less than $k_{B}Tln2$ were registered, while irreversible operations dissipated much more than $k_{B}Tln2$. Measurements of a logically reversible operation on a bit with energy 30 $k_{B}T$ yielded an energy dissipation of 0.01 $k_{B}T$ [89]. Experiments performed with a single colloidal particle trapped in a modulated double-well potential demonstrated that the mean dissipated heat saturated at the Landauer bound in the limit of long erasure cycles [90]. An experiment performed with a colloidal particle in a time-dependent, virtual potential created by a feedback trap also confirmed the Landauer limit [91].

### 2.9. Landauer Limit in the Context of Logical and Thermodynamic Irreversibility

Discussion around the Landauer principle leads to the extremely important distinction between the logic and thermodynamic irreversibility. In order to understand this distinction, we have to start from the separation of the degrees of freedom of the computing device. Some of a computer’s degrees of freedom are used to encode the logical state of the computation process, and these information-bearing degrees of freedom (abbreviated IBDF) are by design sufficiently robust that, within limits, the computer’s logical state evolves deterministically as a function of its initial value, regardless of fluctuations occurring in the environment (i.e., temperature fluctuations) or in the computer’s other non-information-bearing degrees of freedom (NIBDF) [92]. While a computer as an entire physical device (including its power supply and other parts of its environment) may be considered a closed system obeying reversible laws of motion, Landauer noticed that the logical state may evolve irreversibly, with two or more distinct logical states following a single logical successor. Therefore, because Hamiltonian dynamics conserve the fine-grained entropy, the entropy decrease in the IBDF during a logically irreversible operation should necessarily be compensated by an equal or greater entropy increase in the NIBDF and environment. This is the Landauer principle seen in the context of the informational/non-informational degrees of freedom of the computing device [90].

Thus, a clear distinction between thermodynamic and logic reversibility becomes necessary. Following Sagawa, we adopt the following definitions of thermodynamic and logical reversibility: A physical process is thermodynamically reversible if and only if the time evolution of the probability distribution in the process can be time-reversed, where the change in the external parameters is also time-reversed and the signs of the amounts of work and heat are changed [31]. In turn, a computational process $\hat{C}$ is logically reversible if and only if it is an injection. In other words,$\;\hat{C}$ is logically reversible if and only if, for any output logical state, there is a unique input logical state. Otherwise,$\;\hat{C}$ is logically irreversible [31]. The logically irreversible erasure can be performed in a thermodynamically reversible manner in the quasi-static limit. This does not contradict the conventional Landauer principle. The logical reversibility is defined only by the reversibility of the logical states, which is related only to the logical entropy. In contrast, the thermodynamic reversibility is related to the reversibility of the relevant total system (i.e., the whole universe), including the heat bath, and to the total entropy production, as discussed in Section 2. Therefore, these logical and thermodynamic reversibilities are not equivalent in general [31,93]. If the erasure is not quasi-static but is performed with a finite velocity (the Margolus–Levitin limit determines only the minimal time of computation; however, in principle it may be infinite; see Section 2.2), the erasure becomes thermodynamically irreversible. In this specific case, we recover the Landauer bound, as a work, which is necessary for the erasure of one bit of information. For the limit of $ln2k_{B}T$ heat generation per bit to be reached, the thermodynamic process must be reversible. In practice, logical operations are implemented by sub-optimal physical processes and thus are thermodynamically irreversible [93]. However, this irreversibility is not caused by the nature of the logical operation; it is by way of the operation being implemented by a thermodynamically sub-optimal physical process [93]. This is as true for logically irreversible operations as it is for logically reversible operations [93].

### 2.10. Generalization of the Landauer Principle

Generalization of the Landauer principle for logically non-deterministic operations was reported by Maroney [94]. The non-equilibrium quantum Landauer principle was reported [45,95]. The Landauer principle at absolute-zero temperatures was introduced recently; a bound tighter than Landauer that remains nontrivial even in the T→0 was reported [96]. Herrera discussed the Landauer principle in its relation to general relativity [97]. The Landauer principle was applied to the problem of gravitational radiation [97]. The fact that gravitational radiation is an irreversible process entailing dissipation is a straightforward consequence of the Landauer principle and the fact that gravitational radiation conveys information were demonstrated [97]. It should be emphasized that understanding the relativistic extension of the Landauer bound remains an open problem due to the fact that the construction of a relativistic thermodynamics theory is still controversial after more than 110 years of its development. In particular, the problem of the relativistic transformation for temperature remains unsolved [98,99,100,101,102].

### 2.11. Criticism and Objections to the Landauer Principle

The Landauer principle was intensively criticized by J. D. Norton, who argued that since it is not independent of the second law of thermodynamics, it is either unnecessary or insufficient as an exorcism of Maxwell’s demon [103,104,105,106,107,108]. Lairez suggested a counterexample of physical implementation (that uses a two-to-one relation between logic and thermodynamic states) that allows one bit to be erased in a thermodynamic quasi-static manner (i.e., one that may tend to be reversible if slowed down enough) [109]. The Landauer principle was defended in a series of recent papers [110,111,112,113,114]. Witkowski et al. demonstrated an original proof of the Landauer principle that is completely independent of the second law of thermodynamics [112]. Buffoni et al. demonstrated that the Landauer principle, in contrast to widespread opinion, is not the second law of thermodynamics nor is it equivalent to it; in fact, it is a stricter bound [115]. However, the discussion is far from exhausted.

The mass–energy–information equivalence principle, summarized by Equations (19) and (20), was criticized recently [116]. In particular, Lairez argued that (i) isothermal variation in the entropy-rooted part of the free energy of a body (namely, TΔS) is not accompanied by any variation in its mass, (ii) the Landauer–Bennet idea is not a general principle and is only true in a particular case, and (iii) the link between information and energy is valid only for fresh information about a dynamic system. Old information, or information detached from its subject matter, is no longer information and has no value [116]. Thus, the physical groundings of the link between the mass, energy, and information remain debatable and should be clarified. 

### 2.12. The Landauer Principle: Open Questions, Perspectives, and Challenges

In spite of the enormous theoretical and experimental effort spent on the understanding and experimental validation of the Landauer principle, a number of challenging problems remain open.

(i) The exact place of the Landauer principle in the structure of thermodynamics should be clarified. Thermodynamics, in contrast to other fields of physics, enables a completely axiomatic approach, as suggested by Carathéodory [117,118,119]. The second law of thermodynamics was formulated by Carathéodory as follows: “In the neighborhood of any equilibrium state of a system (of any number of thermodynamic coordinates), there exist states that are inaccessible by reversible adiabatic processes.” It seems to be instructive to re-shape the axiomatic thermodynamics with the use of the Landauer principle.
(ii) A relativistic extension of the Landauer principle remains one of the unsolved problems (the problem of the accurate derivation of the relativistic transformation of the temperature also remains open [97,98,99,100,101,102]). This problem is closely related to general cosmology. Calculation of the cosmological constant *Λ* emerging from the Landauer principle was reported [120].
(iii) It is important to implement the Landauer principle in the development of optimal computational protocols, providing minimal dissipation [37,43,121]. Limitations imposed by the Margolus–Levitin limiting principle should be considered (see Section 2.2). The construction of optimal computers remains an open task and is deeply discussed in Ref. [122], in which restrictions imposed on computation by fundamental physical laws are deeply discussed. Ref. [122] is strongly recommended for readers interested in the physics of computation. It was also mentioned that the transfer of entropy and not entropy itself restricts optimal computational protocols [123].
(iv) The philosophical meaning of the Landauer principle should be clarified [124].

## 3. Conclusions

The physical roots, justification, interpretation, controversies, and precise meaning of the Landauer principle remain obscure, in spite of the fact that they have been exposed to turbulent and spirited discussion in the last few decades. The Landauer principle (or the Landauer bound), suggested by Rolf Landauer in 1961, is a physical principle predicting the lower theoretical limit of energy consumption of computation [26,27,28,29]. It states that an irreversible change in information stored on a computer, such as merging two computational paths, dissipates a minimum amount of heat $k_{B}Tln2$ per bit of information to its surroundings. The Landauer principle is discussed in the context of other fundamental physical limiting principles, such as the Abbe diffraction limit, the Margolus–Levitin limit, and the Bekenstein limit [15,16,125]. We demonstrate that the synthesis of the Landauer bound with the Abbe, Margolus–Levitin, and Bekenstein limits quite surprisingly yields the minimum time of computation, which scales as $\tau_{min}\sim \frac{h}{k_{B}T}=\tau_{PB}$ (where *h* and $k_{B}$ are the Planck and Boltzmann constants, respectively), which is exactly the Planck–Boltzmann thermalization time [36,41]. This result leads to a very important conclusion: Decreasing the temperature of a thermal bath will decrease the energy consumption of a single computation, but in parallel, it will slow the computation. The relation between the Landauer bound and the Szilárd minimal engine is discussed. 

The Landauer principle bridges John Archibald Wheeler’s “it from bit” paradigm and thermodynamics [63,75,76]. This bridge yields the mass–energy–information principle, enables calculation of the informational capacity of the universe, and provides a fresh glance at the dark matter problem [66,67,68,69,70,71]. The Landauer principle may serve as a basis for the unification of physical theories, enabling a united, unified approach to the informational content of fields and particles. Generalization of the Landauer principle to quantum and non-equilibrium systems is addressed [44,45,125]. The relativistic aspects of the Landauer principle are discussed. Engineering applications of the Landauer principle in the development of optimal computational protocols are considered [37,43,120]. Experimental verifications of the Landauer principle are surveyed [46,59]. The interrelation between thermodynamic and logical irreversibility is addressed. The non-trivial relationship between the Landauer principle and the second law of thermodynamic is considered [115]. Objections and criticism of the Landauer principle are discussed [103,104,109]. The mass–energy–information equivalence principle was criticized recently [116]. Therefore, a lot of questions related to the Landauer principle and its extensions remain debatable. We conclude that the Landauer principle represents a powerful heuristic principle bridging fundamental physics, information theory, and computer engineering. It is suggested that the Landauer principle may serve as a cornerstone of axiomatic thermodynamics. 

## Data Availability

No new data were created or analyzed in this study. Data sharing is not applicable to this article.

## References

[1] Liu Y.C., Huang K., Xiao Y.-F., Yang L., Qiu C.W. (2021). What limits limits?. Nat. Sci. Rev..

[2] Markov I. (2014). Limits on fundamental limits to computation. Nature.

[3] Hecht E. (2002). Chapter 13 Modern Optics. Optics.

[4] Born M., Wolf E. (1986). Chapter 8 Elements of the theory of diffraction. Principles of Optics.

[5] Zuo R., Liu W., Cheng H., Chen S., Tian J. (2018). Breaking the Diffraction Limit with Radially Polarized Light Based on Dielectric Metalenses. Adv. Opt. Mater..

[6] Einstein A. (1920). Chapter XXII. Relativity: The Special and General Theory.

[7] Fayngold M. (2008). Chapter 2. Special Relativity and How It Works.

[8] Ellis G.F.R., Uzan J.-P. (2005). *c* is the speed of light, isn’t it?. Am. J. Phys..

[9] Anber M.M., Donoghue J.F. (2011). Emergence of a universal limiting speed. Phys. Rev. D.

[10] Landau L.D., Lifshitz E.M. (1977). Chapter 2. Quantum Mechanics: Non-Relativistic Theory.

[11] Cohen-Tannoudji C., Diu B., Laloë F. (1996). Quantum Mechanics.

[12] Bremermann H.J., Yovits M.C., Jacobi G.T., Goldstein G.D. (1962). Optimization through evolution and recombination. Self-Organizing Systems 1962.

[13] Mandelstam L., Tamm I., Bolotovskii B.M., Frenkel V.Y., Peierls R. (1991). The uncertainty relation between energy and time in non-relativistic quantum mechanics. Selected Papers.

[14] Hörnedal N., Sönnerborn O. (2023). Margolus-Levitin quantum speed limit for an arbitrary fidelity. Phys. Rev. Res..

[15] Margolus M., Levitin L.B. (1998). The maximum speed of dynamical evolution. Physica D.

[16] Bekenstein J.D. (1981). Universal upper bound on the entropy-to-energy ratio for bounded systems. Phys. Rev. D.

[17] Anderson J.B., Johnnesson R. (2005). Chapter 1. Understanding Information Transmission.

[18] Ash R.B. (1990). Chapter 1. Information Theory.

[19] Shannon C.E. (1948). A Mathematical Theory of Communication. Bell Syst. Tech. J..

[20] Shannon C.E. (1948). A Mathematical Theory of Communication. Bell Syst. Tech. J..

[21] Ben Naim A. (2017). Shannon’s Measure of information and Boltzmann’s H-Theorem. Entropy.

[22] Ben-Naim A. (2017). Information Theory.

[23] Ben-Naim A. (2008). A Farewell to Entropy: Statistical Thermodynamics Based on Information.

[24] Ben Naim A. (2018). An Informational Theoretical Approach to the Entropy of Liquids and Solutions. Entropy.

[25] Ben-Naim A. (2016). Entropy, the Truth the Whole Truth and Nothing but the Truth.

[26] Landauer R. (1961). Dissipation and heat generation in the computing process. IBM J. Res. Dev..

[27] Landauer R. (1991). Information is physical. Phys. Today.

[28] Landauer R. (1996). Minimal energy requirements in communication. Science.

[29] Bennett C.H., Landauer R. (1985). The fundamental physical limits of computation. Sci. Am..

[30] Parrondo J.M.R., Horowitz J.M., Sagawa T. (2015). Thermodynamics of information. Nat. Phys..

[31] Sagawa T. (2014). Thermodynamic and logical reversibilities revisited. J. Stat. Mech..

[32] Piechocinska B. (2000). Information erasure. Phys. Rev. A.

[33] Landau L.D., Lifshitz E.M. (2011). Statistical Physics.

[34] Kittel C., Kroemer H. (1980). Chapter 3. Thermal Physics.

[35] Bormashenko E. (2019). Generalization of the Landauer Principle for Computing Devices Based on Many-Valued Logic. Entropy.

[36] Hartnoll S.A., Mackenzie A.P. (2022). Colloquium: Planckian dissipation in metals. Rev. Mod. Phys..

[37] Proesmans K., Ehrich J., Bechhoefer J. (2020). Optimal finite-time bit erasure under full control. Phys. Rev. E.

[38] Lee J.S., Lee S., Kwon H., Park H. (2022). Speed Limit for a Highly Irreversible Process and Tight Finite-Time Landauer’s Bound. Phys. Rev. Lett..

[39] Van Vu T., Saito K. (2022). Finite-Time Quantum Landauer Principle and Quantum Coherence. Phys. Rev. Lett..

[40] Ma Y.-H., Chen J.F., Sun C.P., Dong H. (2022). Minimal energy cost to initialize a bit with tolerable error. Phys. Rev. E.

[41] Rolandi A., Llobet M.P. (2023). Finite time Landauer Principle beyond weak coupling. Quantum.

[42] Reeb D., Wolf M.N. (2014). An improved Landauer principle with finite-size corrections. New J. Phys..

[43] Deshpande A., Gopalkrishnanm M., Ouldridge T.E., Jones N.S. (2017). Designing the optimal bit: Balancing energetic cost, speed and reliability. Proc. R. Soc. A.

[44] Lorenzo S., McCloskey R., Ciccarello F., Paternostro M., Palma G.M. (2015). Landauer’s Principle in Multipartite Open Quantum System Dynamics. Phys. Rev. Lett..

[45] Goold J., Paternostro M., Modi K. (2015). Nonequilibrium Quantum Landauer Principle. Phys. Rev. Lett..

[46] Yan L.L., Xiong T.P., Rehan K., Zhou F., Liang D.F., Chen L., Zhang J.Q., Yang W.L., Ma Z.H., Feng M. (2018). Single-Atom Demonstration of the Quantum Landauer Principle. Phys. Rev. Lett..

[47] Peterson J.P.S., Sarthour R.S., Souza A.M., Oliveira I.S., Goold J., Modi K., Soares-Pinto D.O., Céleri L.C. (2016). Experimental demonstration of information to energy conversion in a quantum system at the Landauer limit. Proc. R. Soc. A.

[48] Strasberg P., Schaller G., Brandes T., Esposito M. (2017). Quantum and Information Thermodynamics: A Unifying Framework Based on Repeated Interactions. Phys. Rev. X.

[49] Diana G., Bagci G.B., Esposito M. (2013). Finite-time erasing of information stored in fermionic bits. Phys. Rev. E.

[50] Sagawa T., Ueda M. (2009). Minimal Energy Cost for Thermodynamic Information Processing: Measurement and Information Erasure. Phys. Rev. Lett..

[51] Gavrilov M. (2017). Erasure Without Work in an Asymmetric, Double-Well Potential. Experiments on the Thermodynamics of Information Processing.

[52] Szilard L. (1929). Über die Entropieverminderung in einem thermodynamischen System bei Eingriffen intelligenter Wesen. Z. Phys..

[53] Aydin A., Sisman A., Kosloff R. (2020). Landauer’s Principle in a Quantum Szilard Engine without Maxwell’s Demon. Entropy.

[54] Kim S.W., Sagawa T., De Liberato S., Ueda M. (2011). Quantum Szilard Engine. Phys. Rev. Lett..

[55] Chamberlin R.V. (2024). Small and Simple Systems That Favor the Arrow of Time. Entropy.

[56] Chamberlin R.V. (2015). The Big World of Nanothermodynamics. Entropy.

[57] Hill T.L. (2013). Thermodynamics of Small Systems, Parts I & II.

[58] Bormashenko E., Shkorbatov A., Gendelman O. (2007). The Carnot engine based on the small thermodynamic system: Its efficiency and the ergodic hypothesis. Am. J. Phys..

[59] Koski E.V., Maisi V.F., Pekola J.P., Averin D. (2014). Experimental realization of a Szilard engine with a single electron. Proc. Natl. Acad. Sci. USA.

[60] Koski J.V., Kutvonen A., Khaymovich I.M., Ala-Nissila T., Pekola J.P. (2015). On-Chip Maxwell’s Demon as an Information-Powered Refrigerator. Phys. Rev. Lett..

[61] Bannerman T., Price G.N., Viering K., Raizen M. (2009). Single-photon cooling at the limit of trap dynamics: Maxwell’s demon near maximum efficiency. New J. Phys..

[62] Maruyama K., Morikoshi F., Vedral V. (2005). Thermodynamical detection of entanglement by Maxwell’s demons. Phys. Rev. A.

[63] Wheeler J.A. Information, physics, quantum: The search for links. Proceedings of the 3rd International Symposium on Foundations of Quantum Mechanics in the Light of New Technology.

[64] Herrera L. (2014). The mass of a bit of information and the Brillouin’s principle. Fluct. Noise Lett..

[65] Brillouin L. (1953). The negentropic principle of information. J. Appl. Phys..

[66] Vopson M. (2019). The mass-energy-information equivalence principle. AIP Adv..

[67] Vopson M. (2020). The information catastrophe. AIP Adv..

[68] Vopson M. (2021). Estimation of the information contained in the visible matter of the universe. AIP Adv..

[69] Vopson M. (2022). Experimental protocol for testing the mass–energy–information equivalence principle. AIP Adv..

[70] Vopson M., Lepadatu S. (2022). Second law of information dynamics. AIP Adv..

[71] Vopson M. (2023). The second law of infodynamics and its implications for the simulated universe hypothesis. AIP Adv..

[72] Müller J.G. (2024). Events as Elements of Physical Observation: Experimental Evidence. Entropy.

[73] Müller J.G. (2020). Photon detection as a process of information gain. Entropy.

[74] Müller J.G. (2019). Information contained in molecular motion. Entropy.

[75] Bormashenko E. (2019). The Landauer Principle: Re-Formulation of the Second Thermodynamics Law or a Step to Great Unification?. Entropy.

[76] Bormashenko E. (2020). Informational Reinterpretation of the Mechanics Notions and Laws. Entropy.

[77] Bormashenko E. (2022). Rotating Minimal Thermodynamic Systems. Entropy.

[78] Fixsen D.J. (2009). The Temperature of the cosmic microwave background. Astrophys. J..

[79] Mikhailovsky G.T., Levich A.P. (2015). Entropy, information and complexity or which aims the arrow of time?. Entropy.

[80] Lloyd S. (2002). Computational capacity of the Universe. Phys. Rev. Lett..

[81] Tatum E.T., Seshavatharam U.V.S., Lakshminarayan S. (2015). Flat space cosmology as a mathematical model of quantum gravity or quantum cosmology. Int. J. Astron. Astrophys..

[82] Rubin V.C., Burstein D., Ford W.K., Thonnard N. (1985). Rotation velocities of 16 Sa galaxies and a comparison of Sa Sb and Sc rotation properties. Astrophys. J..

[83] Arkani-Hamed N., Finkbeiner D.P., Slatyer T.R., Weiner N. (2009). A theory of dark matter. Phys. Rev. D.

[84] de Swart J., Bertone G., van Dongen J. (2017). How dark matter came to matter. Nat. Astron..

[85] Bertone G., Hooper D., Silk J. (2005). Particle dark matter: Evidence, candidates and constraints. Phys. Rep..

[86] Dodelson S., Widrow L.M. (1994). Sterile neutrinos as dark matter. Phys. Rev. Lett..

[87] Milgrom M., Sanders R.H. (2003). Modified Newtonian dynamics and the dearth of dark matter in ordinary elliptical galaxies. Astrophys. J..

[88] Bormashenko E. (2020). What Is Temperature? Modern Outlook on the Concept of Temperature. Entropy.

[89] Orlov A.O., Lent C.S., Thorpe C.C., Boechler G.P., Snider G.L. (2012). Experimental Test of Landauer’s Principle at the Sub-k_B_Y Level. Jpn. J. Appl. Phys..

[90] Bérut A., Arakelyan A., Petrosyan A., Ciliberto S., Dillenschneider R., Lutz E. (2012). Experimental verification of Landauer’s principle linking information and thermodynamics. Nature.

[91] Jun Y., Gavrilov M., Bechhoefer J. (2014). High-precision test of Landauer’s principle in a feedback trap. Phys. Rev. Lett..

[92] Bennet C.H. (2003). Notes on Landauer’s principle, reversible computation, and Maxwell’s Demon. Stud. Hist. Philos. Mod. Phys..

[93] Maroney O.J.E. (2005). The (absence of a) relationship between thermodynamic and logical reversibility. Stud. Hist. Philos. Sci. B.

[94] Maroney O.J.E. (2009). Generalizing Landauer’s principle. Phys. Rev. E.

[95] Esposito M., Van den Broeck C. (2011). Second law and Landauer principle far from equilibrium. Europhys. Lett..

[96] Timpanaro A.M., Santos J.P., Landi G.T. (2020). Landauer’s Principle at Zero Temperature. Phys. Rev. Lett..

[97] Herrera L. (2020). Landauer Principle and General Relativity. Entropy.

[98] Farías C., Pinto V.A., Moya P.S. (2017). What is the temperature of a moving body?. Sci. Rep..

[99] Landsberg P., Matsas G.E.A. (2004). The impossibility of a universal relativistic temperature transformation. Phys. A Stat. Mech. Appl..

[100] Papadatos N., Anastopoulos C. (2020). Relativistic quantum thermodynamics of moving systems. Phys. Rev. D.

[101] Papadatos N. (2021). The Quantum Otto Heat Engine with a Relativistically Moving Thermal Bath. Int. J. Theor. Phys..

[102] Güémez J., Mier J.A. (2023). Relativistic thermodynamics on conveyor belt. Phys. Scr..

[103] Norton J.D. (2005). Eaters of the lotus: Landauer’s principle and the return of Maxwell’s demon. Stud. Hist. Philos. Sci. B.

[104] Norton J.D. (2011). Waiting for Landauer. Stud. Hist. Philos. Sci. B.

[105] Lu Z., Jarzynski C. (2019). A Programmable Mechanical Maxwell’s Demon. Entropy.

[106] Leff H., Rex A.F. (2002). Maxwell’s Demon 2 Entropy, Classical and Quantum Information, Computing.

[107] Rex A. (2017). Maxwell’s Demon—A Historical Review. Entropy.

[108] Bub J. (2000). Maxwell’s Demon and the thermodynamics of computation. Stud. Hist. Philos. Sci. B.

[109] Lairez D. (2023). Thermodynamical versus Logical Irreversibility: A Concrete Objection to Landauer’s Principle. Entropy.

[110] Ladyman J., Presnell S., Short A.J., Groisman B. (2007). The connection between logical and thermodynamic irreversibility. Stud. Hist. Philos. Sci. B.

[111] Ladyman J., Robertson K. (2013). Landauer defended: Reply to Norton. Stud. Hist. Philos. Sci. B.

[112] Witkowski C., Brown S., Truong K. (2024). On the Precise Link between Energy and Information. Entropy.

[113] Barnett S.M., Vaccaro J.A. (2013). Beyond Landauer erasure. Entropy.

[114] Lostaglio M., Jennings D., Rudolph T. (2017). Thermodynamic resource theories, non-commutativity and maximum entropy principles. New J. Phys..

[115] Buffoni L., Campisi M. (2022). Spontaneous Fluctuation-Symmetry Breaking and the Landauer Principle. J. Stat. Phys..

[116] Lairez D. (2024). On the Supposed Mass of Entropy and That of Information. Entropy.

[117] Carathéodory C. (1909). Untersuchungen über die Grundlagen der Thermodynamik. Math. Ann..

[118] Pogliani L., Berberan-Santos M.N. (2000). Constantin Carathéodory and the axiomatic Thermodynamics. J. Math. Chem..

[119] Bubuianu I., Vacaru S.I. (2021). Constantin Carathéodory axiomatic approach and Grigory Perelman thermodynamics for geometric flows and cosmological solitonic solutions. Eur. Phys. J. Plus.

[120] Gkigkitzis I., Haranas I., Kirk S. (2013). Number of information and its relation to the cosmological constant resulting from Landauer’s principle. Astrophys. Space Sci..

[121] Gingrich T.R., Rotskoff G.M., Crooks G.E., Geissler P.L. (2016). Near-optimal protocols in complex nonequilibrium transformations. Proc. Natl. Acad. Sci. USA.

[122] Lloyd S. (2000). Ultimate physical limits to computation. Nature.

[123] Prokopenko M., Lizier J. (2015). Transfer Entropy and Transient Limits of Computation. Sci. Rep..

[124] Hemmo M., Shenker O. (2019). The physics of implementing logic: Landauer’s principle and the multiple-computations theorem. Stud. Hist. Philos. Sci. B.

[125] Deffner S. (2020). Quantum speed limits and the maximal rate of information production. Phys. Rev. R.

